# Vitamin C in Stem Cell Biology: Impact on Extracellular Matrix Homeostasis and Epigenetics

**DOI:** 10.1155/2017/8936156

**Published:** 2017-04-20

**Authors:** Cristina D'Aniello, Federica Cermola, Eduardo Jorge Patriarca, Gabriella Minchiotti

**Affiliations:** Stem Cell Fate Laboratory, Institute of Genetics and Biophysics ‘A. Buzzati-Traverso', CNR, 80131 Naples, Italy

## Abstract

Transcription factors and signaling molecules are well-known regulators of stem cell identity and behavior; however, increasing evidence indicates that environmental cues contribute to this complex network of stimuli, acting as crucial determinants of stem cell fate. l-Ascorbic acid (vitamin C (VitC)) has gained growing interest for its multiple functions and mechanisms of action, contributing to the homeostasis of normal tissues and organs as well as to tissue regeneration. Here, we review the main functions of VitC and its effects on stem cells, focusing on its activity as cofactor of Fe^+2^/*α*KG dioxygenases, which regulate the epigenetic signatures, the redox status, and the extracellular matrix (ECM) composition, depending on the enzymes' subcellular localization. Acting as cofactor of collagen prolyl hydroxylases in the endoplasmic reticulum, VitC regulates ECM/collagen homeostasis and plays a key role in the differentiation of mesenchymal stem cells towards osteoblasts, chondrocytes, and tendons. In the nucleus, VitC enhances the activity of DNA and histone demethylases, improving somatic cell reprogramming and pushing embryonic stem cell towards the naive pluripotent state. The broad spectrum of actions of VitC highlights its relevance for stem cell biology in both physiology and disease.

## 1. Introduction

l-Ascorbic acid (vitamin C (VitC)) was extensively studied over the last century because it plays an essential role for proper folding and deposition of collagen proteins, which are the most abundant proteins in the human body and have a strong impact on the composition/structure/biomechanical features of the extracellular matrix (ECM). Human cells are unable to synthesize VitC, and therefore, it must constantly be restored through the diet. Indeed, under VitC deprivation, human cells are unable to generate and maintain healthy tissues, in particular those rich in collagens such as the skin, bones, and cartilage, and VitC deficiency in humans causes scurvy, a complex syndrome characterized by generalized ECM dissolution and tissue disintegration. It is only until recently that ECM homeostasis was considered the unique molecular mechanism influenced by VitC availability. In the last years, the use of cutting-edge technologies (next-generation sequencing and advanced microscopy) to study stem cell biology have broadened enormously our knowledge of VitC activities. Specifically, VitC has emerged as a key regulator of stem cell identity/behavior, influencing pluripotency, self-renewal, and differentiation. VitC enhances somatic cell reprogramming, that is, the generation of induced pluripotent stem cells (iPSCs), and pushes embryonic stem cells toward a naive state of pluripotency by modulating the cellular epigenetic profile [1–3]. The strong biological, biotechnological, and medical significance of VitC-dependent molecular mechanisms become even more relevant taking into account another key VitC-dependent cellular modification, that is, collagen hydroxylation, which is the most abundant posttranslation modification found in the human proteoma. In this review, we will focus on the recent progress made on the influence of VitC on stem cell biology and its implications for regenerative medicine.

## 2. Vitamin C Metabolism and Functions

**Figure 1:**
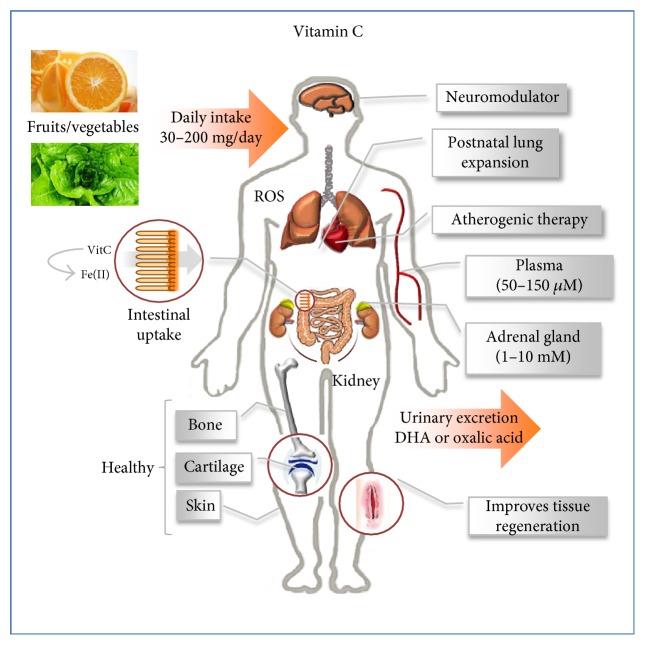
Vitamin C metabolism and activities. Vitamin C, in humans, must be introduced by daily intake through diet. It plays crucial roles both for the proper function of healthy organs and tissues and for tissue repair and regeneration. VitC may act as a scavenger against reactive oxygen species (ROS) and as a chelator, for example, iron metabolism. Both VitC and its catabolic product, dehydroascorbate (DHA), are excreted through urine.

VitC is a naturally occurring small carbohydrate (3-keto-l-gulofuranlactone) synthesized by a two-step reaction mainly from l-galactose or d-galacturonic acid in green plants [4]. Humans are unable to synthesize VitC due to the lack of the l-gulonolactone oxidase (GLO) enzyme and therefore are strictly dependent on an exogenous source of VitC. Its level is maintained in a range between micromolar in the blood plasma (~50 *μ*M) and millimolar (~1–10 mM) inside the cells (Figure 1), with the highest levels found in pituitary and adrenal gland cells where it is accumulated through the activity of highly specific transport systems encoded by the *SLC23A1* and *SLC23A2* genes, also known as *SVCT1* and *SVCT2* [5–7]. VitC is continuously catabolized by oxidation to dehydroascorbate (DHA), which in turn is converted into oxalic acid [8]. The main route of elimination of VitC and DHA is urinary excretion (Figure 1). Oxalate is one of the major end products of VitC breakdown in humans, and this may cause accumulation of calcium oxalate stones and nephrocalcinosis; thus, susceptible people should avoid systematic ingestion of vitamin C supplements [9].

### 2.1. ROS Neutralizer and Iron Chelator

VitC is considered the most relevant naturally occurring reducing substance [10]. Inside the cells, VitC cooperates to maintain the intracellular redox balance. VitC reduces reactive oxygen species (ROS), including superoxide anion (O_2_^−1^), hydroxyl radical (OH^−^), singlet oxygen (O_2_^∗^), and hypochlorous acid (HClO), which are generated during mitochondrial oxidative phosphorylation (aerobic ATP generation). ROS regulate several signaling pathways involved in pluripotency, including MAPKs, ERKs, p38MAPKs, JNKs, and MAPK phosphatases. Interestingly, VitC inhibits NFkB activation in human cell lines (U937, HL-60, and MCF-7) and in primary cells (HUVEC) in a dose-dependent manner [11]. ROS inactivation results in VitC oxidation to dehydroascorbic acid (DHA), which in turn alters cellular homeostasis. DHA can be reduced to VitC (DHA → VitC) by enzymatic and nonenzymatic activities involving glutathione and homocysteine, which regenerate/recycle VitC [12, 13]. Besides its role as antioxidant, VitC exerts a chelator activity; indeed, by reducing ferric to ferrous (Fe^+3^ → Fe^+2^) iron and by generating soluble iron complexes, VitC efficiently enhances the absorption of nonheme iron at the intestine level [14–17]. The chromaffin granule cytochrome b_561_ (CGCyt b_561_) and the duodenal Cyt b_561_ (DCyt b_561_) are transmembrane oxidoreductases [18, 19], which contribute to recycle VitC from DHA and enhance iron absorption. Indeed, while CGCyt b_561_ catalyzes the transfer of electrons from cytoplasmic VitC to intravesicular DHA (DHA → VitC), DCyt b_561_ transfers electrons from cytoplasmic VitC to Fe^+3^ ions in the intestinal lumen, thus generating soluble Fe^+2^ ions which are eventually taken up by the cells through a Fe^2+^ transporter [20, 21]. As recently reviewed [22], VitC impacts on iron metabolism also stimulate ferritin synthesis, inhibit lysosomal ferritin degradation and cellular iron efflux, and induce iron uptake from low-molecular weight iron-citrate complexes.

### 2.2. Enzymatic Cofactor/Enhancer

**Figure 2:**
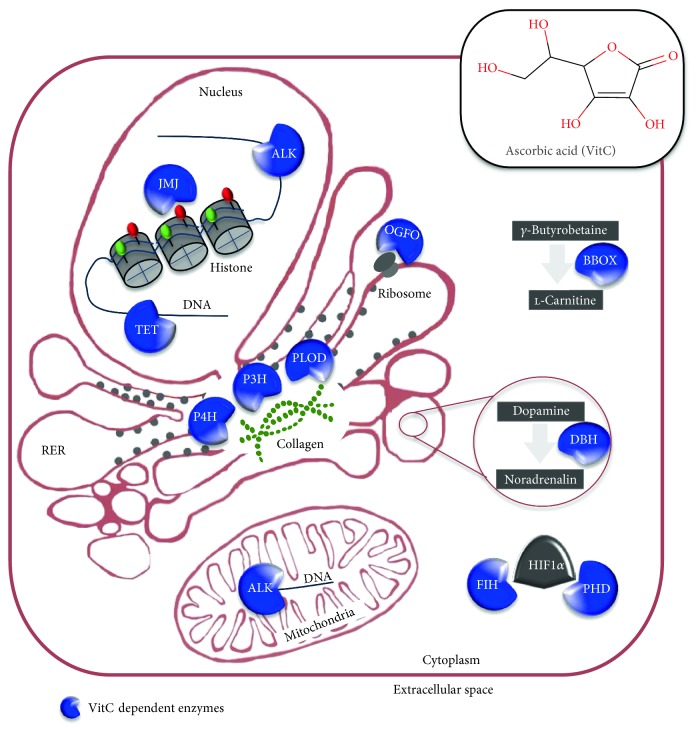
Localization and functions of VitC-dependent mono- and dioxygenase enzymes. ALK: RNA and DNA demethylase family; JMJ: jumonji histone demethylases; TET: ten-eleven translocation DNA demethylases; OGFO: 2-oxoglutarate- and Fe^2+^-dependent oxygenase; PLOD: procollagen-lysine_*α*-KG_5-dioxygenases; P3H: collagen prolyl 3-hydroxylase; P4H: collagen prolyl 4-hydroxylases; BBOX1: *γ*-butyrobetaine dioxygenases; DBH: dopamine beta-monooxygenase; PHD: HIF-prolyl hydroxylase; FIH: factor inhibiting HIF.

Besides its role as antioxidant, VitC is essential for the activity of a family of mono- and dioxygenases enzymes (EC 1.14.11) by providing the electrons required to keep the prosthetic metal ions in the reduced/active form, specifically Cu^+1^ (cuprous) for the monoxygenases and Fe^+2^ (ferrous) for the dioxygenases [23, 24]. In mammals, VitC-dependent oxygenases catalyze the hydroxylation of DNA, peptides/proteins, and lipids as well as a wide variety of small molecules. For instance, VitC is the cofactor of the *γ*-butyrobetaine dioxygenase (BBOX1), which catalyzes the final step of L-carnitine biosynthesis (Figure 2). This enzyme is involved in the transport of fatty acids inside the mitochondria for *β*-oxidation and modulates osteogenesis and chondrogenesis in adipose- and bone marrow-derived stem cells [25]. Similarly, VitC is required for the conversion of the neurotransmitter dopamine to noradrenalin (dopamine beta-monooxygenase (DBH)), the metabolism of tyrosine and the amidation of peptide hormones. VitC-dependent enzymes also include the prolyl hydroxylases that regulate the hydroxylation and thus the degradation of the hypoxia-inducible factor (HIF). Conversely, 5-(hydroxymethyl)-2-furfural (5-HMF) stabilizes HIF protein by reducing VitC level [26]. Of note, VitC enhances the activity of the asparaginyl hydroxylase factor inhibiting HIF-1 (FIH-1), which is an important suppressor of hypoxia-inducible factor (HIF) activity [27]. Interestingly, HIF regulates stem cell pluripotency and self-renewal controlling specific signaling pathways and transcription factors [28]. Most remarkably, VitC enhances the activity of a specific class of RNA and/or DNA demethylases, that is, the human AlkB homologue enzymes (VitC/Fe^+2^/*α*KG-dependent dioxygenases) [29, 30], including ABH1 (or ALKBH1), which catalyzes the demethylation of 3-meC in DNA and RNA [31]; ABH2 (ALKBH2), which catalyzes the oxidative demethylation of 1-methyladenine [32]; ABH3 (ALKBH3), which repairs methylated RNA [33]; and FTO, which demethylates 3-methylthimidine (3-meT) and 3-methyluracil [34], whose variants were found associated with obesity in both children and adults [35]. Another class of VitC/Fe^+2^/*α*KG-dependent demethylases has recently gained great attention, due to their key role in somatic cell reprogramming, specifically, the jumonji (JHDM, KDM) family, which are engaged in histone demethylation (chromatin-modifying oxygenases) and the DNA demethylases of the ten-eleven translocation (TET) family [36–40]. Finally, the P4HA and PLOD enzymes belong to the same family of VitC/Fe^+2^/*α*KG-dependent dioxygenases and catalyze the hydroxylation of collagen proline and lysine residues. Thus, VitC activity is essential for the activity of key epigenetic enzymes as well as for the conversion of procollagen to collagen. Moreover, it has recently been reported that VitC stimulates the iron-mediated nonenzymatic conversion of the oncometabolite *α*-hydroxyglutarate (2-HG) into *α*-ketoglutarate [41]. 2HG is a competitive inhibitor of *α*-ketoglutarate dioxygenases [42]. Therefore, VitC influences the epigenetic signature, the metabolism (fatty acid catabolism), and the microenvironment (collagen/ECM composition) of the cells, thus pointing to a key role of VitC availability in shaping cell identity/behavior.

### 2.3. VitC Localization

By acting as antioxidant, VitC is required in all the subcellular compartments but particularly inside the oxidative organelles (mitochondria, endoplasmic reticulum, and peroxisomes). Indeed, by acting as an enzymatic cofactor, its requirement depends on the enzymes' subcellular localization (Figure 2). For instance, histone and DNA demethylases are all located in the nucleoplasm. Specifically, AlkB human homologues (ALBH or ALKBH enzymes) are located in the nucleoplasm and inside the mitochondria where they catalyze the demethylation of 3-meC residues on DNA and RNA, respectively [31]. Collagen prolyl/lysyl hydroxylases are localized in the ER lumen [43], HIF prolyl hydroxylases (PHDs) in the cytosol, and dopamine *β*-monooxygenase and peptidylglycine *α*-hydroxylating monooxygenase (PHM) in the synaptic and secretory vesicles. Since VitC is a water-soluble molecule, specific transport systems should be activated to keep optimal VitC concentrations in each subcellular compartment. Two families of transporters are associated with VitC transport in human cells, the sodium- (Na^+1^-) coupled ascorbic acid transporters (SVCTs; SLC23), which are highly specific for reduced VitC, and the members of the glucose transporter (GLUT; SLC2) family, some of which also transport DHA. SVCT2 transporter colocalizes with the protein disulfide isomerase (PDI) and marks both ER and mitochondrial membranes. Interestingly, the embryonic brain cortex of SVCT2 KO mouse mutants produces significant lower levels of several neurotransmitters, including dopamine and norepinephrine [44] and, furthermore, SVCT2 knockdown mitochondria inefficiently transport VitC [45]. Finally, homozygous Slc23a1^−/−^ mice die at birth with respiratory failure and intraparenchymal brain hemorrhage [46]. SLC23A1 is essential for renal reabsorption and hepatic accumulation of VitC but not for its intestinal transport [47]. Interestingly, specific polymorphisms in the sodium-dependent VitC transporter 2 gene increase the risk of incident of acute coronary syndrome in women, but not in man [48]. So far, the transporters that facilitate VitC accumulation in the intraluminal ER have not been characterized at a molecular level. However, defects in the subcellular distribution of VitC may cause diseases and aging. For instance, impaired mitochondrial uptake of VitC/DHA and thus a VitC shortage in the mitochondrial matrix should provoke a defective removal of ROS. Indeed, it has recently been hypothesized that VitC may be channelled from the nucleoplasm to the ER lumen through the ER subdomain nuclear envelope [43], which may eventually reduce the level of VitC in the nucleus and thus its availability for the epigenetic enzymes. A similar subcellular redistribution of VitC may occur as a consequence of a rapid and massive accumulation of collagen synthesis, for instance upon stimulation with transforming growth factor-*β* (TGF*β*). Thus, it is interesting to hypothesize that changes in VitC levels in the different subcellular compartments could impact on the redox status, the epigenetic signature, and the ECM composition and eventually modify cell behavior. In this respect, it is important to take into account that VitC subcellular distribution likely depends on the expression levels and binding affinities of the different VitC-dependent enzymes and carriers; however, our knowledge is still limited and this issue needs to be further studied [43].

## 3. VitC-Dependent Regulation of ECM/Collagen Homeostasis

The extracellular matrix (ECM) is a complex mix of fibrillar proteins and polysaccharides synthesized and secreted by the cells in the extracellular space. Indeed, with the exception of the neural extracellular matrix [49], the ECM has a fibrillar structure in most tissues and provides a structural scaffold to the surrounding cells that is essential for tissue/organ morphogenesis, as well as for their regeneration after injury. Collagens are the most abundant proteins in the ECM and thus the most abundant proteins in mammals (~30% of total protein mass) [50] making up 90% of the bone tissue [51]. Collagens are crucial for the development and maintenance of the skin, cartilage, tendons, ligaments, and of the blood vessels and are deposited in the ECM where they generate supramolecular assemblies/complexes contributing to the shape and mechanical properties of tissues, such as the tensile strength in the skin and the ligament resistance to traction [52]. VitC thus impacts on ECM homeostasis by regulating collagen synthesis and maturation.

### 3.1. Collagen Synthesis

VitC promotes the transcription of collagen genes and/or increases the stability of collagen mRNA in many different cell lines, including human skin fibroblasts [53, 54], PAT cells [55], and murine 3T3-L1 preadipocytes [56]. Also, profibrotic cytokines of the TGF*β* family stimulate collagen synthesis, especially in wound healing and fibrotic diseases [57]. Interestingly, activation of the TGF*β* pathway enhances collagen synthesis and reduces collagen degradation in different cell lines, including human mesenchymal stem cells [58], human marrow stromal cell [59], human dermal fibroblasts [60–62], glomerular mesangial cells [63], lung alveolar epithelial cells [64], and vascular smooth muscle cells (VSMCs) [65], thus resulting in fibrosis/ECM accumulation. In line with these findings, in human dermal fibroblasts, several collagen-coding genes, including *COL1A2*, *COL3A1*, *COL6A1*, and *COL6A3*, have been identified as TGF*β*/SMAD3 targets in human dermal fibroblasts [66]. Moreover, vitamin D-induced reduction of intestinal fibrosis has been associated with the inhibition of the canonical TGF*β*/SMAD3 pathway [67]. In renal epithelial cells, TGF*β* regulates collagen deposition by recruiting mTOR kinase (through noncanonical TGF*β* pathway) [47, 68]. Interestingly, mTOR regulates HIF-1*α*, which in turn is controlled by VitC and regulates the transcription of *COL1A2* (collagen I *α*2) gene. Indeed, knockdown of DEPTOR, an mTOR signaling inhibitor, induces collagen expression; conversely knockdown of RAPTOR, which conversely is a positive regulator of mTOR, inhibits collagen expression. TGF*β* can increase collagen synthesis also by inducing the cleavage of the cAMP response element-binding protein 3-like 1 (CREB3L1) transcription factor [69]. Of note, collagen synthesis may be induced also independently of the TGF*β* signaling as described during hypoxia-dependent mesenchymalization of human lung epithelial A549 cell line [70].

### 3.2. Collagen Prolyl and Lysyl Hydroxylases

Collagens are synthesized as procollagen molecules, which are subjected to numerous posttranslational modifications, that is, hydroxylation of l-pro and l-lys residues, glycosylation of l-lys and hydroxylysine residues, and sulfation of tyrosine (Tyr) residues (see [71]). Collagen synthesis also requires the activity of specific posttranslational enzymes that are inactivated by the formation of the collagen triple helix. First, collagen hydroxylation is required for the correct folding of procollagen polypeptide chains into stable triple helical molecules. Collagen lysyl hydroxylases, also known as procollagen-lysine_*α*-KG_5-dioxygenases, encoded by *PLOD1*, *PLOD2*, and *PLOD3* genes, are VitC-dependent enzymes that catalyze the lysine hydroxylation [72, 73]. Collagen prolyl 4-hydroxylases (P4Hs) are VitC-dependent enzymes that catalyze the proline hydroxylation in collagens. Collagen prolyl hydroxylation involves three isoforms of the P4HA subunit (P4HA1, P4HA2, and P4HA3) that form A_2_B_2_ tetramers with P4HB and eventually P4H1, P4H2, and P4H3 holoenzymes, respectively. Collagen prolyl hydroxylation is the major posttranslational modification in the human proteoma [74]. In the absence of P4H activity, the procollagen molecules are unable to exit the ER [75, 76]. Interestingly, it has been previously reported that PH activity is induced early during wound healing and that its induction is associated with the onset of collagen biosynthesis and deposition [77]. Increasing evidence suggest that VitC-dependent collagen hydroxylation positively correlates with tumor aggressiveness. Recently, it has been shown that silencing of P4HA2 (collagen prolyl 4-hydroxylase *α* subunit 2) expression inhibits proliferation and suppresses the most aggressive phenotype of breast cancer cells in vivo [78]. Of note, treatment with the P4H inhibitor ethyl 3,4-dihydroxybenzoate induces a similar phenotype [79]. Interestingly, P4HA1 is highly expressed in aggressive prostate cancer and it is essential for in vivo cancer progression [80].

### 3.3. Collagen Signaling

Collagen precursors (procollagen/~300 nanometers in length) are synthesized in the endoplasmic reticulum (ER), packaged into transport vesicles, and delivered to Golgi cisternae where fibrillogenesis occurs. The resulting collagen fibrils are the longest (up to millimeters in length) and the largest protein polymers in vertebrates [51, 81]. Collagens are degraded in the extracellular microenvironment through the activity of zinc-dependent endopeptidases, that is, the matrix metalloproteinases (MMPs), which are the key enzymes involved in physiological (development and tissue repair) and pathological (tumorigenesis and metastasis) processes [82]. Collagens participate in cell-matrix interactions acting as functional ligands of several receptor families including glycoprotein VI (GPVI), inhibitory leukocyte-associated immunoglobulin-like receptor-1 (LAIR-1), Endo180 (urokinase-type plasminogen activator-associated protein), integrins, and dimeric discoidin receptors (DDR1 and DDR2) [83–85]. Of note, DDR receptors are powerful inhibitors of collagen deposition (fibrillogenesis) [86]. These collagen-receptor interactions modulate cell growth, differentiation, and migration. Of note, *β*(1) integrins are required for correct embryoid body formation and cardiac fate specification and differentiation of induced pluripotent stem cells [87].

### 3.4. Influence of ECM in Tissue Generation, Regeneration, and Cancer

VitC influences ECM composition/structure, and it is now evident that the mechanical features of the ECM influence normal and cancer stem cell behavior [88–93]. For instance, while soft substrates (0.6 kPa polyacrylamide gels coated with of type-1 collagen) sustain embryonic stem cell (ESC) self-renewal [94], rigid/stiff substrates induce differentiation/lineage specification of mesenchymal stem cells (MSCs) [95]. ECM structure also impacts on tumor cells' behavior. Indeed, breast cancer malignancy is associated with ECM stiffening [88], and increased collagen deposition and collagen fiber diameter, through ROCK activation, drive epidermal hyperplasia [90]. Interestingly, VitC-dependent collagen prolyl and lysyl hydroxylases are key regulators of breast cancer metastasis [79, 96, 97]. In particular, HIF-1-mediated induction of VitC-dependent PLOD2 enzyme is required for deposition of fibrillar collagen and increase tumor stiffness [96]. Recently, the use of soft fibrin gels allowed the isolation of a subpopulation of highly tumorigenic melanoma cancer cells, named tumor-repopulating cells (TRCs) [98]. Of note, TRC self-renewal relies on a specific epigenetic modification, that is, VitC-dependent demethylation of the histone 3 lysine 9 (H3K9) [99]. Indeed, silencing of H3K9 demethylases inhibits TRC self-renewal [99].

Besides its involvement in tumor cell progression, it is well known that ECM is a key determinant within the stem cell niche, influencing also normal stem cell behavior and identity. Indeed, several signals and factors produced by the ECM have been reported to integrate with other signaling pathways and transcription factors, thus finely modulating stem cell proliferation, self-renewal, and cell fate decisions both in vitro and in vivo. In particular, integrin receptors respond to ECM signals, regulating stem cell differentiation in early embryogenesis. Indeed, integrin *β*1 is essential for inner-cell mass development [100], and laminin-deficient embryos are unable to undergo epiblast differentiation and cavitation [101]. In line with these findings, type-1 collagen (collagen-1) facilitates mESC self-renewal in vitro [102]. Moreover, VitC-dependent collagen synthesis is essential for the induction of ESC cardiac differentiation, which in turn is impaired by two inhibitors of collagen synthesis (l-2-azetidine carboxylic acid and cis-4-hydroxy-d-proline) [103]. Of note, ECM signaling also influences self-renewal of various somatic stem cells, safeguarding epidermal stem cell compartment [104], neural stem cell maintenance and behavior [105], and hematopoietic stem cell self-renewal and differentiation [106]. Furthermore, ECM is crucial for the skeletal system development, function, and repair, impacting on different stem cell types such as osteoblasts/osteoclasts involved in bone remodeling [107] and mesenchymal stem cells [108], chondrocytes and tenocytes [109], and muscle stem cells (for a review, see [110]). Based on these observations, VitC availability might influence stem cell phenotype/behavior by regulating ECM stiffness and homeostasis and thus playing a crucial role for tissue function.

## 4. VitC as Epigenetic Modifier

Epigenetic changes are important regulators of gene expression both in development and in diseases. Among them, addition of a methyl group to the C5 position of cytosine on DNA and to the lysine at different positions (K4, 9, 27, and 36) of histone 3 (H3) represents the major and best characterized epigenetic modification. VitC impacts the epigenetic signature of the cells by promoting the activity of the Fe^2+^ and *α*KG-dependent dioxygenases involved in DNA and histone demethylation.

### 4.1. VitC and DNA Demethylation

DNA methylation is catalyzed by DNA methyltransferases (DNMTs) and plays a pivotal role in modulating transcription activity and cellular identity. In mammals, it mostly involves the cytosine residues of CpG dinucleotides. Global DNA demethylation occurs early during embryo development, at the preimplantation stages, that is, before the inner cell mass (ICM) specification [111, 112]. Conversely, a widespread DNA remethylation occurs during gastrulation and leads to lineage restriction and loss of pluripotency. Aberrant DNA methylation is a hallmark of cancer [113, 114], and global DNA hypomethylation is associated with poor prognosis in tumor patients [115]. Global changes in DNA methylation patterns have been associated with cardiovascular diseases, essential hypertension, inflammation, autoimmune diseases, and infections [116–120]. DNA demethylation depends on the catalytic activity of the VitC/Fe^+2^/*α*KG-dependent TET (ten-eleven translocation) enzymes, which convert 5-methylcytosine (5-mC) into 5-hydroxymethylcytosine (5-hmC). A further TET-mediated oxidation of 5-hmC to 5-formylcytosine (5-fC) and to 5-carboxylcytosine (5-caC), together with the activation of the base excision repair mechanism, lead to a complete demethylation process [38]. Of note, blocking VitC entry into the cells by phloretin and/or preventing (knocking down) the expression of Tet genes (*Tet1*, *Tet2*, and *Tet3*) by short interference RNAs (siRNA) significantly reduces VitC-dependent 5-hmC induction [121]. Moreover, treatment of cells with glutathione, an antioxidant agent, does not alter the level of 5-hmC; thus, indicating that VitC-dependent induction of 5-hmC is not due to its activity as an antioxidant, thus supporting a key role of VitC as a cofactor for TET DNA dioxygenases. Interestingly, in hepatocellular carcinoma cells, VitC enhances the demethylating effect of 5-Azacytidine (a DNA methyl transferase inhibitor), inducing the expression/activity of Tet enzymes and increasing the levels of 5-hmC [122].

### 4.2. VitC and Histone Demethylation

The transfer of a methyl group to lysine and arginine residues of histone proteins is the principal epigenetic modification that occurs on histones and is part of the epigenetic mechanisms that controls stem cell homeostasis. This reaction is catalyzed by histone methyltransferases, which, as DNMTs, use S-adenosylmethionine as donor of methyl groups. While for DNA methylation, only one methyl group is added to cytosine, for histones, up to three or two methyl groups can be added to lysine and arginine, respectively. The resulting mono-, di-, and trimethylated residues can either promote or silence chromatin, depending on the methylated residue. As for DNA methylation, also histone methylation is a reversible process, which depends on the activity of histone demethylases, such as the VitC/Fe^+2^/*α*-KG dependent demethylases, that is, the JmjC domain-containing histone demethylases. Thus, VitC enhances the activity of several JmjC domain-containing histone demethylases, inducing histone demethylation and contributing to establish the epigenetic signature of the cells. For instance, VitC counteracts H3K9 and H3K36 and DNA methylation induced by exogenously provided l-Proline in ESCs [123].

### 4.3. VitC-Dependent Dioxygenases in Stemness, Fibrosis, and Cancer

VitC availability has been reported to be crucial for stem cell identity and plasticity. Increased VitC availability (100 *μ*g/ml) in mouse ESCs promotes widespread DNA demethylation through TET activity [3] and pushes ESCs towards a naive state of pluripotency, which can be placed between the naive/2i and FBS/LIF cultures [1]. VitC induces extensive DNA hypomethylation also in human embryonic stem cells (hESCs) [124]. VitC maintains the methylated pattern and regulates the expression of the imprinted Dlk1-Dio3 cluster in ESCs and acquisition of full pluripotency [125]. Indeed, VitC promotes TET activity (DNA demethylation) and enhances the generation of mouse and human-induced pluripotent stem cells (iPSCs) leading to the transcriptional activation of pluripotency gene network [121, 126, 127]. Similarly, VitC promotes histone demethylation, reducing the level of H3K9me3 and enhancing the pre-iPSC to iPSC transition [2, 128, 129]. Accordingly, mouse embryonic fibroblasts (MEFs) upon KD of the Tet enzymes fail to undergo the mesenchymal to epithelial transition necessary for the reprogramming process [128, 130, 131]. In line with these findings, VitC activity exerts a key role in the early stages of embryo development, as suggested by several developmental defects induced by VitC deficiency. Indeed, although the knowledge of the mechanism is still limited, several evidence indicate a key role of VitC-dependent activity of DNA and histone demethylases during early embryonic development. Indeed, VitC-induced Tet3 activity is required for epigenetic reprogramming of the zygotic paternal DNA and for the subsequent demethylation of the maternal DNA [132]. Moreover, VitC promotes a second round of demethylation in primordial germ cells (PGC) [133].

Epigenetic changes induced by VitC are important modulators of cell identity and are also considered hallmarks of several pathological conditions. In particular, different types of cancers, including leukemia, melanoma, colorectal adenoma, and gastric cancers, show reduced levels of 5-hmC, which may alter the normal regulation of gene expression and lead to malignant transformation. Interestingly, mutations that result in decreased expression and/or altered function of TET enzymes, as well as mutations in *SVCT* genes that reduce the normal uptake of VitC and thus the level of 5-hmC, have been described in human cancers [134, 135]. Interestingly, restoring the levels of 5-hmC could at least in part decrease malignancies [134]. While the role of epigenetic alterations, that is, reduced 5-hmC levels, has been mostly implicated and studied in cancers, their relevance in other diseases remains still poorly understood. A growing interest is emerging in the context of fibrotic diseases. Although different studies support the idea that the acquisition of the profibrotic characteristics in different pathologies is associated with epigenetic modifications that control changes in gene expression profiles, the impact of DNA methylation on the acquisition of the profibrotic features is still under debate [136].

## 5. VitC in Stem Cells

Scurvy is characterized by a generalized tissue disintegration, dissolution of intercellular ECM, which induces an excessive proliferation of undifferentiated cells and a reversion to a primitive form of the tissue [137]. This suggests a putative role of the VitC-collagen/ECM integrity on the control of precursor cells' proliferation. Interestingly, stromal changes (ECM depolymerization) also arise at the invasive front or in the proximity of invading neoplastic cells of aggressive tumors, where VitC mostly accumulates [138]. Furthermore, several evidence indicate that VitC stimulates the proliferation of different mesenchyme-derived cell types including osteoblasts, adipocytes, chondrocytes, and odontoblasts [103, 108, 139–146], as well as the proliferation of immunologically relevant T cells [147] and hyalocytes (eye vitreous cells) [148]. Depending on the concentration used, the incubation time, and the cell type analyzed, VitC can inhibit and/or induce stem cell proliferation and/or the differentiation (Table 1). For instance, VitC safeguards the differentiation potency of bone marrow-derived multipotent *mesenchymal stem cells* (MSCs) stimulating their in vitro proliferation [139]. In these cells, VitC stimulates ECM secretion (collagen and glycosaminoglycan). *Adipocyte stem cells* (ASCs) are a heterogeneous population of MSCs that can differentiate into adipocytes, osteoblast, and chondrocytes. L-Ascorbate-2-phosphate (A2-P or Asc-2-P, 250 *μ*M) enhances proliferation of human ASCs and induces the formation of an ASC sheet displaying abundant extracellular matrix (ECM) deposition [149]. Of note, A2-P-treated ASCs maintain both high levels of expression of pluripotency-associated transcription factors *Sox2*, *Oct4*, and *Nanog* and their differentiation capabilities along adipogenic and osteogenic (mesoderm) lineages. This is highly relevant since ASCs cultivated in the absence of VitC tend to lose their stemness and pluripotency. Moreover, A2-P-treated ASCs display enhanced *hepatogenic* (endoderm) and *neurogenic* (ectoderm) transdifferentiation capabilities under specific conditions [149]. Most relevantly, collagen synthesis appears to be required for VitC activity [149]. VitC covalently coupled to a methyl methacrylate polymer enhances the proliferation of bone marrow-derived human MSCs [143]. VitC also promotes proliferation of *cardiac progenitor cells* [150]. A mix of micronutrients, including VitC, transferrin, glutathione, selenite, and ethanolamine, sustains the in vitro expansion/self-renewal of mouse *intestinal stem cells* (ISCs) [151]. ISCs are located at the base of the intestinal crypts and, under appropriate stimuli, are able to divide and differentiate into mature epithelial cells. VitC (40 *μ*g/ml) promotes the proliferation of *spermatogonial stem cells* (SSCs) and reduces the generation of ROS while it induces the expression of the *Bcl-2* (antiapoptotic) gene [152]. Of note, this activity is highly specific of VitC.

### 5.1. VitC Positively Regulates the Pluripotency Genes

VitC, like retinoic acid (vitamin A) and calciferol (vitamin D), modulates gene expression [153]. VitC induces *Nanog* expression in ESCs and safeguards pluripotency [154]. Similarly, VitC enhances the expression of *Nanog* in mouse teratocarcinoma-derived EC cells (F9) and inhibits the retinoic acid-induced differentiation of EC cells through the Janus kinase/signal transducer and activator of transcription (JAK/STAT) signaling pathway [155]. At mechanistic level, VitC induces STAT2 phosphorylation, which in turn activates *Nanog* transcription [155]. VitC enhances telomerase activity in *periodontal ligament stem cells* (PDLSCs) and upregulates the expression of extracellular matrix type-I collagen, fibronectin, integrin *β*1, and the stem cell markers *Oct4*, *Sox2*, and *Nanog* as well as osteogenic markers *Runx2*, *Alp*, and *Ocn* [156]. In hESCs, VitC induces the expression of CD30, which is a biomarker for malignant cells in Hodgkin's disease and embryonal carcinoma cells, through a dramatic loss of DNA methylation of a CpG island in the CD30 promoter [157]. Furthermore, VitC is a competitive inhibitor of adenylate cyclase [158] and thus could repress the expression of the genes controlled by the cAMP-dependent pathway [159]. The molecular mechanism(s) involved in VitC-dependent induction of pluripotency markers are not well understood and deserve further investigation.

### 5.2. Impact of VitC on Stem Cell Differentiation

**Figure 3:**
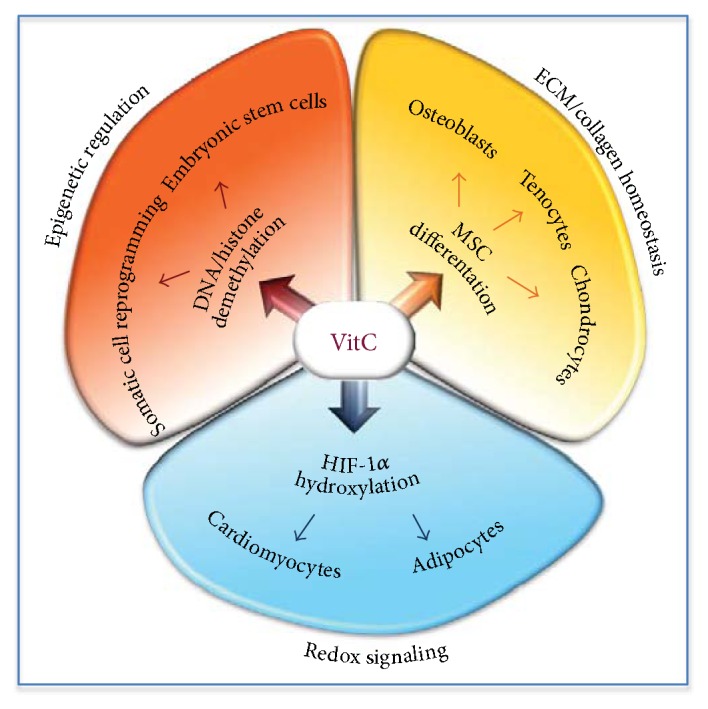
Molecular mechanisms underlying VitC activity on stem cells. VitC-dependent regulation of epigenetic modifications, ECM remodelling and redox balance, control embryonic stem cell self-renewal/proliferation, somatic cell reprogramming, and stem cell differentiation.

A vast body of evidence supports the notion that supplemental VitC improves the differentiation and maintenance of mesenchyme-derived connective tissues, including adipose tissue, cartilage, bone, and blood (Figure 3). For instance, a combination of VitC and beta-glycerophosphate promotes differentiation of mouse ESCs toward the osteoblast lineage [160]. VitC also enhances osteoblastic differentiation of adipocyte-derived progenitor cells [161]. Moreover, a combination of staphylococcal enterotoxin C and VitC (50 *μ*g/ml) promotes osteoblastic differentiation in bone marrow-derived MSCs [162]. Furthermore, supplemental VitC (250 *μ*M) enhances osteogenesis from umbilical cord blood-derived MSCs, whereas higher VitC concentrations (500 *μ*M) reduces MSC proliferation [163]. Low levels of VitC induce osteogenic differentiation of MG-63 osteosarcoma cell line, whereas at higher doses induce apoptosis [164]. These findings are relevant considering that impaired MSC bone differentiation potential induces osteosarcoma [164]. VitC also enhances osteoclastogenesis in ESCs [145], whereas a cocktail including VitC (25 *μ*g/ml), rosiglitazone, insulin, T3, dexamethasone, and indomethacin, significantly increases ESC adipocyte differentiation [165]. VitC enhances differentiation of mesencephalic precursor cells into dopaminergic neurons [166, 167]. It has been shown that VitC promotes epidermal keratinocyte differentiation and that downregulation of protein kinase C activity has abolished the prodifferentiating effect of VitC [141]. A mix of TGF*β* and VitC (30 *μ*M) promotes differentiation of bone marrow-derived MSCs into *smooth muscle cells* (SMCs) [168, 169]. VitC improves cardiac differentiation of mouse ESCs [170]. Of note, VitC promotes cardiac differentiation only when supplemented to the culture medium in a specific time window (day 2–6 differentiation) [150]. Remarkably, VitC addition at the early phases of the differentiation process overcomes the loss of cardiomyocyte differentiation ability in fibroblast growth factor receptor 1 (Fgfr1) knockout (Fgfr1^−/−^) mESCs [171]. The molecular mechanisms underlying VitC-dependent regulation of stem cell differentiation process are largely unknown. The antioxidant activity of VitC is unlikely to be the primary underlying mechanism given that other antioxidants do not induce the same phenotypic transition/differentiation process. Most likely, VitC may influence stem cell differentiation by modulating DNA and histone demethylation. Accordingly, Tet1 and Tet2 double-knockout mESCs display developmental defects when injected in the mouse blastocyst [172]. Unlike wild-type mESCs, *Tet1* knockdown mESCs generate teratomas containing predominately immature glandular tissues (endoderm) surrounded by stromal cells (mesoderm) and trophoblastic giant cells, thus indicating that *Tet1* is essential for cell lineage specification [173]. Moreover, Tet1/2/3 triple knockout results in impaired differentiation potential of mESCs [174]. Furthermore, *Tet2* silencing in hematopoietic stem cell impairs differentiation and alters hematopoiesis [175], whereas it induces neuroectoderm differentiation in ESCs [176]. Conversely, Tet2 overexpression sustains mESC self-renewal while it impairs differentiation [177].

### 5.3. VitC in Somatic Cell Reprogramming

It is well known that the chemical composition of the medium strongly influences the epigenetic and biological properties of iPSCs [178]. Indeed, only under specific growth conditions, iPSCs acquire an ESC-like gene expression profile and epigenetic signature. In line with its ability to maintain pluripotency, supplemental VitC improves mouse and human somatic cell reprogramming [2] (Figure 3). Interestingly, addition of VitC (50 ng/ml) prevents aberrant DNA hypermethylation of the imprinted *Dlk1-Dio3* gene cluster and improves the generation of fully pluripotent iPSCs [125]. VitC treatment enhances the expression of pluripotency markers (*Oct4*, *Sox2*, and *Klf4*) during reprogramming of porcine somatic cells through nuclear transfer [179]. Of note, other antioxidants, such as N-acetylcysteine (NAC) and vitamin E, are unable to similarly improve cell reprograming, thus suggesting that this activity does not rely, at least primarily, on the role of VitC as antioxidant [170]. Several reports have led to the conclusion that VitC promotes the generation of induced pluripotent stem cells (iPSCs) through the activation of both histone-demethylating dioxygenases (JMJ) and TET DNA-demethylating enzymes [131]. A putative role of VitC as enhancer of the collagen synthesis/maturation has not been analyzed. Here, we speculate that somatic cells such as fibroblast may suffer VitC starvation in their nucleus, due to an excessive utilization at the level of ER (collagen synthesis). Exogenously, added VitC may thus compensate this nuclear VitC starvation, thus enhancing DNA and histone demethylation and the expression of pluripotency genes. This intriguing hypothesis may explain at least in part VitC effects on reprograming and deserves further investigation.

## 6. Biotechnological and Medical Applications

VitC is used for the in vitro production of mammalian embryos [180]. Indeed, in a narrow window of concentrations, VitC improves oocyte maturation and the subsequent development of preimplantation embryos. For instance, added at 50 *μ*g/ml, but not at 100 *μ*g/ml, VitC improves porcine oocyte maturation by increasing the cleavage rates and the total cell numbers per blastocyst and by reducing apoptotic cell death [181]. Of note, it has been established that treatment with VitC increases the pregnancy rate in pigs [179]. Furthermore, in vitro culture of ovarian follicles is an emerging tool for fertility preservation. It has been reported that VitC (50 *μ*g/ml) supplementation significantly enhances the survival of early stage (primary) ovarian follicles (<80 *μ*m) cultured in alginate hydrogels, avoiding the breakdown of the follicular basement membrane [182]. In correlation, at the cellular level, VitC upregulates the expression of extracellular matrix (ECM) and cell adhesion molecules [182]. In adult humans, VitC accumulates in the brain, acting as antioxidant and as neuromodulator for acetylcholine and noradrenaline release [183]. In correlation, recent studies have suggested that supplemental VitC could be beneficial for the treatment of neurodegenerative disorders [184]. As a reducing agent, VitC is used for the treatment of methemoglobinemia [185], an autosomal recessive disorder provoked by the deficiency of methemoglobin reductase (OMIM 250800). VitC is extensively used to attenuate the symptoms of common cold [186–190]. In correlation, it has been reported that DHA, the oxidized form of VitC, inhibits the multiplication of viruses of three different families: herpes simplex virus type 1 (HSV-1), influenza virus type A, and poliovirus type 1, perhaps at the step of nucleocapsid formation occurring inside the Golgi cisternae of infected cells [191]. It has been proposed that VitC may contribute to maintain a healthy skin by altering the gene expression profile of dermal fibroblasts [192]. Indeed, VitC has modified significantly the expression of more than 250 genes in vitro cultured human dermal fibroblasts [192]. The transcriptome modifications involve mainly genes related with regulation of the cell cycle and/or mitosis, DNA replication and/or repair, lipid and glucose metabolism, cytoskeleton and ECM remodeling, and collagen biosynthesis [192]. It has also been observed that an increased intake of VitC improves tissue regeneration after surgical trauma, myocardial infarction, and thermal burns. The effect of VitC on cancer prevention and/or regression has been extensively studied, but the results are controversial and beyond the scope of this review. It has recently been shown that high concentrations of dehydroascorbate (DHA), that is, the oxidized form of VitC, induce oxidative stress and cell death in cancer cells [193], thus refurbishing the interest in the use of supranutritional doses of VitC as anticancer. Nonetheless, a high-dose VitC regimen as anticancer therapy has some contraindications [194] and raises some significant questions [195]. Finally, the effects of VitC on the behavior/identity of cancer stem cells have not been studied but would deserve attention.

## 7. Conclusions

An increasing number of reports reveal that VitC impacts on stem cell plasticity/identity and that this largely depends on its ability to sustain the activity of several Fe^+2^/*α*KG dioxygenase enzymes, which catalyze the hydroxylation (oxidation) of different biological substrates located in specific cellular compartments. Specifically, in the nucleus, VitC modulates the activity of several DNA and histone hydroxylases, whereas in the endoplasmic reticulum, VitC acts as cofactor of collagen hydroxylases. Therefore, VitC is able to modify simultaneously both the epigenetic/gene expression profile and the extracellular matrix (microenvironment) of stem cells. In some cases, the effect of VitC activity appears to be dose dependent within a physiological concentration range. However, whether or not VitC-dependent reactions may rely on specific VitC concentrations requires further investigation, due to the current limited knowledge of the key molecular and biochemical features (i.e., expression profiles, molecular interactions, and kinetic parameters) of the enzymes and/or transporters in the different cellular compartments.

## Figures and Tables

**Table 1:** 

Cell type	Effect of exogenously supplied vitamin C	Concentration	Ref.
ASCs (adipocyte stem cells)	↑ Proliferation	250 *μ*g/ml	[149]
hMSCs (human mesenchymal stem cells)	↑ Proliferation	50 *μ*g/ml	[143]
CPC (cardiac progenitor cells)	↑ Proliferation	10–250 *μ*g/ml	[150]
ISCs (intestinal stem cells)	↑ Proliferation/self-renewal	10–250 *μ*M	[151]
Caprine SSCs (spermatogonial stem cells)	↑ Proliferation	40 *μ*M	[152]
Cord blood-derived MSCs (mesenchymal stem cells)	↓ Proliferation	500 *μ*M	[163]
TBV2 ESCs	↑ Naive state of plurypotency	50–150 *μ*g/ml	[1]
ESCs	↑ Naive state of plurypotency	10–500 mM	[3]
Ovarian follicles	↑ Survival	50 *μ*g/ml	[182]
Porcine oocytes	↑ Maturation	50 *μ*g/ml	[181]
J1 ESCs	↑ Pluripotency marker expression	50 *μ*g/ml	[154]
Periodontal ligament stem cells (PDLSCs)	↑ Telomerase activity	20–50 *μ*g/ml	[156]
Bone marrow-derived MSCs (mesenchymal stem cells)	↑ Osteoblastic differentiation	50 *μ*g/ml	[162]
Cord blood-derived MSCs (mesenchymal stem cells)	↑ Osteogenesis	250 *μ*M	[163]
ESCs	↑ Osteoclastogenesis	50 *μ*g/ml	[145]
hMSCs (human mesenchymal stem cells)	↑ Tenogenesis	50 *μ*g/ml	[93]
E14 ESCs	↑ Adipocyte differentiation	25 *μ*g/ml	[165]
C6R8 mESCs	↑ Cardiogenesis	10–100 *μ*M	[170]
Fgfr1^−⁄−^ R1 mESCs	↑ Rescue of cardiomyocyte differentiation	10 *μ*M	[171]
iPSCs	↑ Cardiogenesis	10–250 *μ*g/ml	[150]
HaCaT cells	↑ Epidermal keratinocyte differentiation	1 mM	[141]
Bone marrow-derived MSCs (mesenchymal stem cells)	↑ Smooth muscle cell differentiation (SMCs)	30 *μ*M	[169]; [168]
Mesencephalic precursor cells	↑ Dopaminergic neuron differentiation	100 *μ*M	[167]; [166]
Pre-iPSCs from MEF	↑ Reprogramming	25–50 *μ*g/ml	[2]
B-cells	↑ Reprogramming	50 ng/ml	[125]
Porcine somatic cells	↑ Reprogramming	50 *μ*g/ml	[179]
MEFs	↑ Reprogramming	25–50 *μ*g/ml	[131]
